# Supplementary figures and images for: Growth parameters and responses of green algae across a gradient of phototrophic, mixotrophic and heterotrophic conditions
Source: PeerJ. 2022 Jul 21;10:e13776. doi: 10.7717/peerj.13776 (PMC9308967; doi:10.7717/peerj.13776)

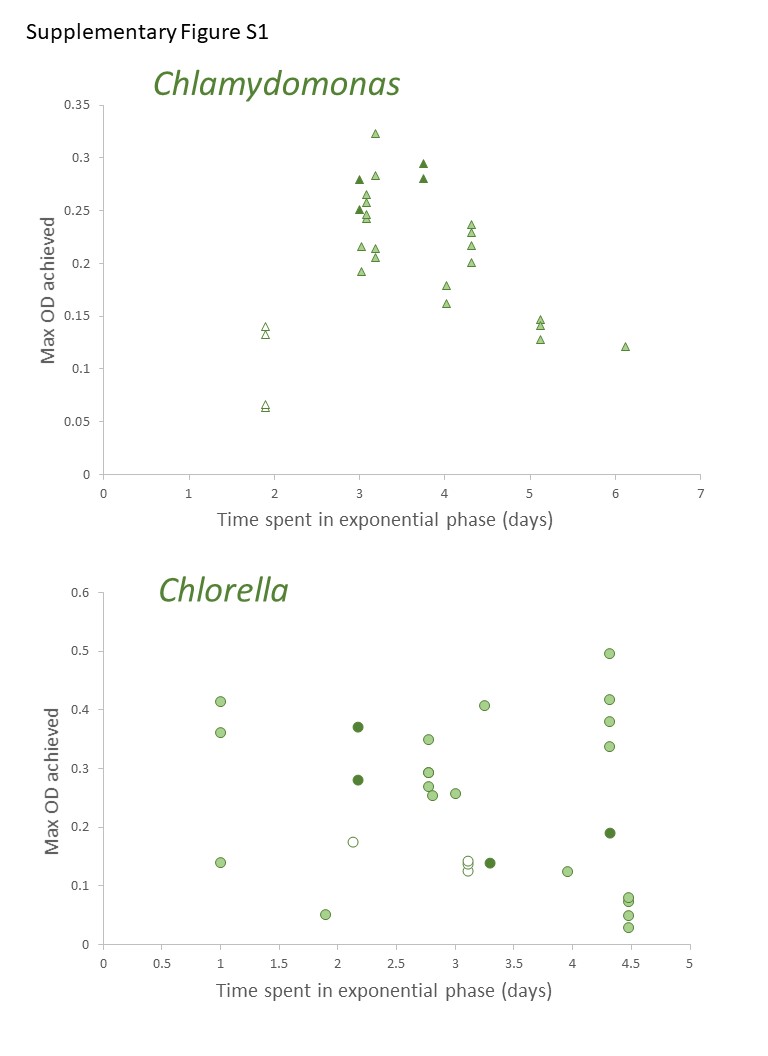

Supplement: Supplemental Information 1 — Relationship between maximum biomass achieved (max OD) and the culture time spent in exponential phase, based on OD680 measurements of cultures. Points indicate values for each of four replicate cultures per treatment (see Fig. 2). Symbols for heterotrophic cultures are filled with white, photoautotrophic cultures are dark green, mixotrophic are light green. There was no clear relationship between the time a culture spent in exponential phase and how high a biomass accumulated in the culture, although in Chlamydomonas, some inverse relationship is suggested for light-grown cultures (light and dark green). [file peerj-10-13776-s001.jpg]
